# Decade-long insights into transperineal prostate biopsy in a West China population: temporal trend, targeted and repeat biopsies, and pathological characterization: a comparative study – retrospective cohort

**DOI:** 10.1097/JS9.0000000000002122

**Published:** 2024-10-25

**Authors:** Jinge Zhao, Diming Cai, Yuntian Chen, Jindong Dai, Xiang Tu, Junru Chen, Guangxi Sun, Xingming Zhang, Jiayu Liang, Xu Hu, Jin Yao, Haoyang Liu, Junjie Zhao, Mengni Zhang, Xueqin Chen, Zhenhua Liu, Ling Nie, Ni Chen, Pengfei Shen, Hao Zeng

**Affiliations:** a Department of Urology, Institute of Urology, West China Hospital, Sichuan University, Chengdu, People’s Republic of China; b Department of Ultrasound, West China Hospital, Sichuan University, Chengdu, People’s Republic of China; c Department of Radiology, West China Hospital, Sichuan University, Chengdu, People’s Republic of China; d Department of Pathology, West China Hospital, Sichuan University, Chengdu, People’s Republic of China

Highlights

- In the west China region represented by our center, a steady annual increase in PBx was observed, with positive rates climbing to ~55%.
- Relative to Western counterparts, prostate cancer patients in west China demonstrated higher M1 stage frequencies, accompanied by older ages and higher baseline PSA levels in M0 cases.
- A possible reduction in systematic biopsy cores, if MRI-targeted biopsy is employed, could maintain accurate detection rates of clinically significant cancers.
- Nonadenocarcinoma prostate cancers, although rarer, were identified with unique clinical characteristics and a higher prevalence in metastatic cases.
- Repeat biopsies tended to diagnose less aggressive cancers, suggesting implications for managing patient follow-up and biopsy strategies.

Since Berringer’s initial report of prostate biopsy (PBx) in 1922^1^, technological advancements in this procedure have significantly evolved. Leveraging PBx records spanning over 10 years and involving 10 378 PBx from 10 038 cases, this study aims to shed light on temporal trends in PBx positivity, the evolving clinical profiles of prostate cancer (PCa) patients, and the differences in clinicopathological characteristics of PCa between Western and Asian populations. We also assessed the real-world effectiveness of MRI-targeted biopsy (MRI-TBx) and repeat biopsy, and characterized the prevalence and characteristics of nonadenocarcinoma PCa. The overall study design of the current study is shown in Figure S1 (Supplemental Digital Content 1, http://links.lww.com/JS9/D523).

Between 2011 and 2022, 10 038 men received transperineal PBx, involving 9733 with a single biopsy and 305 with multiple, totaling 10 378 biopsies. Overall, 5548 PCa were identified, including 86.5% clinically significant PCa (csPCa) and 13.5% insignificant prostate cancer (Ins-PCa). The prebiopsy characteristics of the total cohort are presented in Table S1 (Supplemental Digital Content 2, http://links.lww.com/JS9/D524). An annual increase in PBx was witnessed to over 1000 since 2019, with a positive rate stabilizing at around 55% (Fig. 1A). Figures S2, S3 (Supplemental Digital Content 1, http://links.lww.com/JS9/D523) presents the PBx positivity across the entire cohort and men with prebiopsy PSA < and ≥20 ng/ml. Age, prostate-specific antigen (PSA), prostate volume, and PSA density (PSAD) were strongly correlated with PCa diagnoses (Fig. S4, S5, Supplemental Digital Content 1, http://links.lww.com/JS9/D523). Over time, there was a gradual decrease in both the median age and PSA among men undergoing PBx and diagnosed with PCa (Fig. 1B). International Society of Urological (ISUP) grading revealed grade group (GG) 5 as the most common in PCa patients, followed by GG2 and GG3, with GG4 and GG1 being less frequent (Fig. 1C). The incidence of M1 PCa has been gradually decreasing, dropping below 30% in 2022 (26%) (Fig. 1D). Remarkably, M0 PCa patients showed significant decreases in age and PSA, alongside a rising prevalence of ISUP GG1-3, while M1 patients exhibited no such changes and GG5 remained predominant (Fig. S6A,B, Supplemental Digital Content 1, http://links.lww.com/JS9/D523).

**Figure 1:**
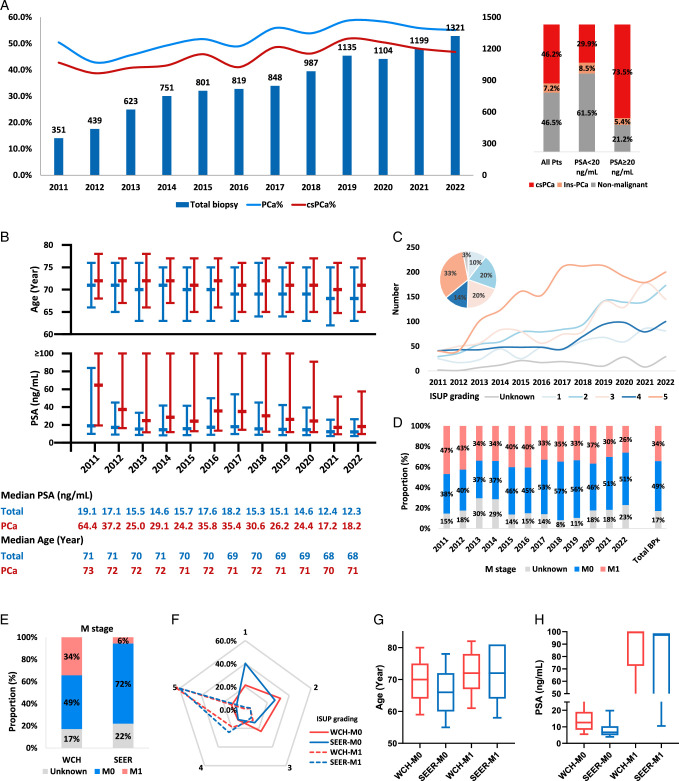
Temporal trends in prostate biopsy positivity and clinical characteristics of prostate cancer. A. The annual changes in the total number of PBx, PCa%, and csPCa%, and the proportion of csPCa, Ins-PCa in the total cohort and men with PSA < and ≥20 ng/ml. B. The temporal changes in the median and IQR of pre-PBx age and PSA for men receiving PBx and those diagnosed with PCa. C. The temporal changes of ISUP grading of PCa. D. The temporal changes of M stage of PCa. E–H. A comparative analysis of the clinicopathological characteristics between PCa patients from our center and those concurrently documented in the SEER database, focusing on M stage (E), ISUP grading (F), age at diagnosis (G), and initial PSA levels (H). csPCa, clinically significant prostate cancer; ISUP, International Society of Urological Pathology; Ins-PCa, insignificant prostate cancer; IQR, interquartile range; PBx, prostate biopsy; PCa, prostate cancer; SEER, Surveillance, Epidemiology, and End Results.

We compared PCa patients at our center and in the SEER database (Fig. 1E–H). The incidence of M1 PCa was significantly higher at our center, 34%, as opposed to 6% in the SEER database (Fig. 1E). Notably, the ISUP grading scores distribution for M1 PCa was comparable between both cohorts, while for nonmetastatic PCa, PCa patients in SEER database harbored more favorable ISUP grading against patients in our institution (Fig. 1F). Particularly, the SEER database showed 40.3% of patients in GG 1, almost doubled that at our center (21.4%). Similarly, M1 patients from both cohorts demonstrated similar pre-PBx ages and PSA, whereas nonmetastatic PCa patients at our center were older (median: 70 vs. 66 years, *P*<0.001) and had higher PSA levels (median: 12.34 vs. 5.9 ng/ml, *P*<0.001) (Fig. 1G,H).

The application of multiparametric MRI (mpMRI) before biopsy significantly improved PCa detection (Fig. S7, Supplemental Digital Content 1, http://links.lww.com/JS9/D523). A strong link was found between higher Prostate Imaging Reporting and Data System (PI-RADS) scores and greater csPCa probability (Fig. 2A). For men with PSA <20 ng/ml, using a PI-RADS score ≥3 to predict csPCa demonstrated high sensitivity (98.80%) and negative predictive value (94.29%), but lower specificity (10.33%) and positive predictive value (36.52%), consistent with the PROMIS study findings^2^. A model combining PSAD and PI-RADS scores was proposed for accurate csPCa risk prediction^3^. Herein, we validated this model in our cohort with baseline PSA <10 or 20 ng/ml (Fig. 2B–E), supporting its effectiveness in csPCa detection. Notably, while the original model categorized a PI-RADS score 3 with PSAD >0.15 as intermediate-high risk, our data suggested a higher PSAD threshold (>0.2), correlating with csPCa positivity rates of 18.5 and 23.1% for prebiopsy PSA levels <10 and 20 ng/ml, respectively.

**Figure 2:**
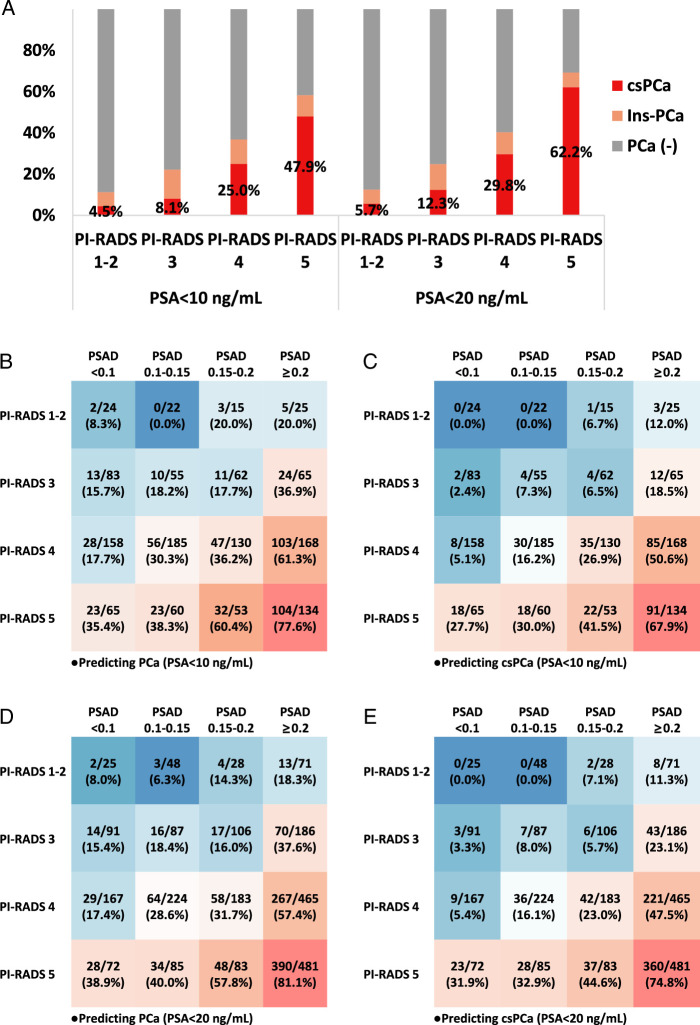
Predictive significance of PI-RADS score and PSAD on prostate cancer. A. The predictive significance of PI-RADS scoring on csPCa and Ins-PCa for patients with PSA <10 ng/ml and PSA <20 ng/ml. B, C. The predictive significance of the PSAD and PI-RADS scoring combined model on PCa (B) and csPCa (C) for men with PSA <10 ng/ml. D, E. The predictive significance of the PSAD and PI-RADS scoring combined model on PCa (D) and csPCa (E) for men with PSA <20 ng/ml. csPCa, clinically significant prostate cancer; Ins-PCa, insignificant prostate cancer; PSAD, prostate-specific antigen density; PI-RADS, Prostate Imaging-Reporting and Data System; PSA, prostate-specific antigen; PCa, prostate cancer.

In our cohort, 450 men with PSA <20 ng/ml had MRI-TBx along with a 12-core SBx. The positive rate of MRI-TBx cores (36.0%) was significantly higher than SBx cores (15.4%) (Fig. 3A, B). Combining SBx and MRI-TBx numerically improved PCa detection compared to SBx alone, and identified about 10% more PCa than MRI-TBx alone (Fig. 3C). Besides, MRI-TBx found a higher tumor proportion than SBx (median: 60% vs. 20%, *P*<0.001, Fig. 3D). csPCa was detected in 186/450 (41.3%) of cases with combined MRI-TBx and SBx, with 121/186 (65.1%) identified by both methods (Fig. 3E). csPCa was found exclusively by MRI-TBx in 24/186 (12.9%) and by SBx in 23/186 (12.4%). MRI-TBx also led to higher ISUP grading in 12 csPCa patients, versus 6 for SBx. Resultingly, adding MRI-TBx to SBx significantly improved csPCa detection and accurate ISUP scoring (186/450 [41.3%] vs. 150/450 [33.3%], *P*=0.016).

**Figure 3:**
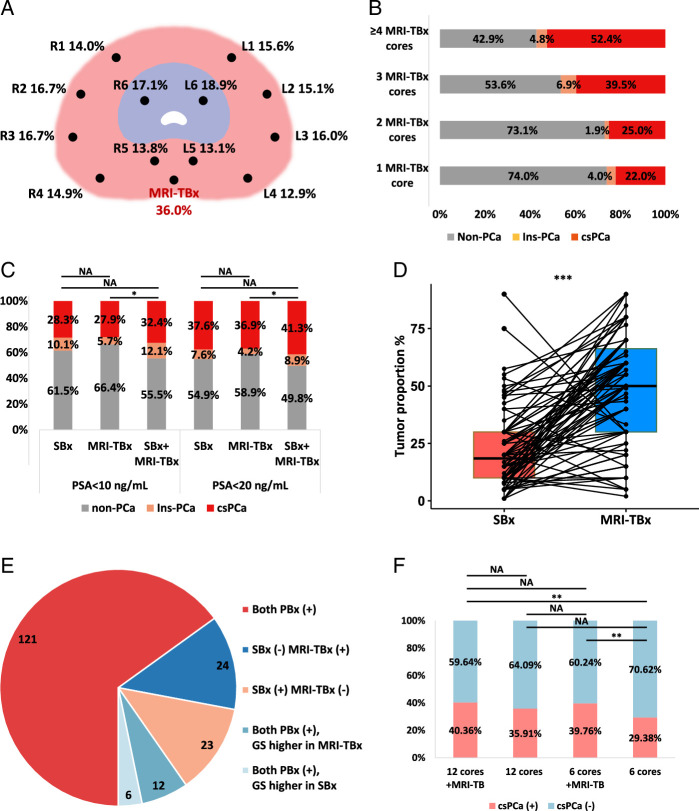
The effectiveness of MRI-targeted prostate biopsy in the real-world setting. A. Core-level positivity rates for men undergoing 12-core SBx and MRI-TBx. B. The detection rate of csPCa and Ins-PCa for different numbers of cores in MRI-TBx. C. The comparison of the detection rate of csPCa and Ins-PCa using SBx, MRI-TBx, and the combination of both. D. The comparison of tumor proportion between cores obtained through SBx and those acquired through MRI-TBx, based on data from 76 patients with available tumor proportion information. E. A comparison of tumor detection rate between SBx and MRI-TBx in patients with PCa. F. The comparison of the csPCa detection rate using 12-core SBx+MRI-TBx, 12-core SBx, 6-core SBx+MRI-TBx, and 6-core SBx in men with PSA <20 ng/ml. *<0.05; **<0.01; ***<0.001. csPCa, clinically significant prostate cancer; Ins-PCa, insignificant prostate cancer; ISUP, International Society of Urological Pathology; MRI-TBx, MRI-targeted prostate biopsy; PCa, prostate cancer; PSA, prostate-specific antigen; SBx, systematic biopsy.

We explored the potential of reducing SBx cores by adding MRI-TBx in men with PSA <20 ng/ml. Specifically, we assessed the effectiveness of 2–3 MRI-TBx cores combined with a 6-core SBx. The 6-core SBx involved random sampling of three cores from each prostate lobe (Fig. S8, Supplemental Digital Content 1, http://links.lww.com/JS9/D523). Analysis of 1000 random cases revealed a median csPCa detection rate of 39.76% with this combination, comparable to the 40.36% rate using a 12-core SBx with MRI-TBx (Fig. 3F), suggesting that fewer SBx cores may be feasible when using MRI-TBx.

We also delved into the prevalence and clinical features of less prevalent nonadenocarcinoma (non-AC) PCa. Overall, non-AC PCa was detected in 1455 men (Fig. S9A, Supplemental Digital Content 1, http://links.lww.com/JS9/D523). In decreasing order, these were: intraductal carcinoma of the prostate (IDC-P, 17.9%), prostatic ductal adenocarcinoma (DA, 5.8%), adenocarcinoma of neuroendocrine differentiation (NED, 5.3%), Signet ring cell carcinoma (SRCC, 0.7%), and small cell neuroendocrine carcinoma (SCC, 0.5%). Non-AC PCa were less common in localized cases, comprising about 15%, but were more prevalent in over half of the metastatic cases. Patients with non-AC PCa had similar ages at diagnosis (Fig. S9B, Supplemental Digital Content 1, http://links.lww.com/JS9/D523). Except for those with SCC, who had PSA levels similar to adenocarcinoma-only patients, those with other non-AC types, especially IDC-P, showed significantly higher initial PSA levels (Fig. S9C, Supplemental Digital Content 1, http://links.lww.com/JS9/D523). Most non-AC PCa patients were classified as ISUP grading ≥4, with over 50% of IDC-P, NED, SCC, and SRCC in ISUP GG 5 (Fig. S9D, Supplemental Digital Content 1, http://links.lww.com/JS9/D523). Non-AC cases had a higher occurrence of M1 stage and elevated PI-RADS scores compared to pure adenocarcinoma (Fig.S9E,F, Supplemental Digital Content 1, http://links.lww.com/JS9/D523). Our prior research linked IDC-P presence with worse outcomes in advanced PCa^4,5^. This study further analyzed IDC-P detection rates across different patient characteristics, revealing a steady increase in IDC-P incidence from the M0 stage, through the locally advanced stage, to the M1 stage. Besides, younger men were more likely to harbor IDC-P (Fig. S10, Supplemental Digital Content 1, http://links.lww.com/JS9/D523).

Three hundred five men underwent repeat PBx after an initial negative result (Fig. S11A, Supplemental Digital Content 1, http://links.lww.com/JS9/D523). Ultimately, 30.5% of cases were diagnosed with PCa. The interval between initial and repeat PBx was longer for men with a positive repeat PBx (33.1-mo vs. 19.7-mo, *P*=0.004, Fig. S11B, Supplemental Digital Content 1, http://links.lww.com/JS9/D523). Compared to initial PBx diagnoses, repeat PBx diagnoses showed less aggressive tumors. Specifically, the csPCa rate was significantly lower in repeat PBx (18.2% vs. 47.2%, *P*<0.001, Fig. S11C, Supplemental Digital Content 1, http://links.lww.com/JS9/D523), with fewer positive cores (median: 16.7% vs. 66.7%, *P*<0.001, Fig. S11D, Supplemental Digital Content 1, http://links.lww.com/JS9/D523). PCa diagnosed in repeat PBx also had a lower rate of high-grade cancer (ISUP GG 3–5, 40.2% vs. 69.1%, *P*<0.001, Fig. S11E, Supplemental Digital Content 1, http://links.lww.com/JS9/D523) and less frequent M1 stage (14.0% vs. 34.5%, *P*<0.001, Fig. S11F, Supplemental Digital Content 1, http://links.lww.com/JS9/D523) against those diagnosed initially.

Collectively, this study analyzed a decade of PBx data from a major Asian cohort, offering vital insights into the changing epidemiology and clinical variations of PBx in China. It highlighted key differences in PCa presentations between China and Western populations; evaluated the efficacy of modern biopsy techniques, including PI-RADS scoring, MRI-TBx, and repeat PBx in real-world settings; and additionally, uncovered distinct clinical features of non-AC PCa types. These insights are essential for future PCa diagnosis and treatment strategies, particularly for China populations.

## Ethical approval

The study received approval from the Medical Ethics Committee of West China Hospital, Sichuan University (Reference number: 2021-1703).

## Consent

Due to the study’s retrospective design, the requirement for written informed consent was waived.

## Source of funding

This work was supported by the National Natural Science Foundation of China (NSFC 82203110, 82172785, and 81974398), 1.3.5 project for disciplines of excellence, West China Hospital, Sichuan University (ZYJC21020), Science and Technology Support Program of Sichuan Province (2021YFS0119), Clinical and Translational Medicine Research Project, Chinese Academy of Medical Sciences (2022-12M-C&T-B-098), Beijing Bethune Charitable Foundation (mnzl202002, mnzl202007), and Postdoctoral Research and Development Fund of West China Hospital of Sichuan University (2023HXBH024).

## Author contribution

J.Z., N.C., P.S., and H.Z.: study concept or design; J.Z., D.C., Y.C., J.D., X.T., J.C., G.S., X.Z., J.L., X.H., J.Y., H.L., J.Z., M.Z., X.C., Z.L., and L.N.: data collection; J.Z., D.C., Y.C., J.D., and X.T.: data analysis or interpretation and writing the paper; J.Z., D.C., N.C., P.S., and H.Z.: critically revised the paper.

## Conflicts of interest disclosure

The authors declare that they have no financial conflict of interest with regard to the content of this report.

## Research registration unique identifying number (UIN)

The research was retrospectively registered (NCT06366308).

## Guarantor

Hao Zeng.

## Data availability statement

Datasets in this study are available upon reasonable request.

## Provenance and peer review

This is not an invited paper.

## Supplementary Material
