# Supplementary material for: Decade-long insights into transperineal prostate biopsy in a West China population: temporal trend, targeted and repeat biopsies, and pathological characterization: a comparative study – retrospective cohort
Source: Int J Surg. 2024 Oct 25;111(1):1636–41. doi: 10.1097/JS9.0000000000002122 (PMC11745631; doi:10.1097/JS9.0000000000002122)
Supplement: Supplementary file 1 [file js9-111-1636-s001.pdf]

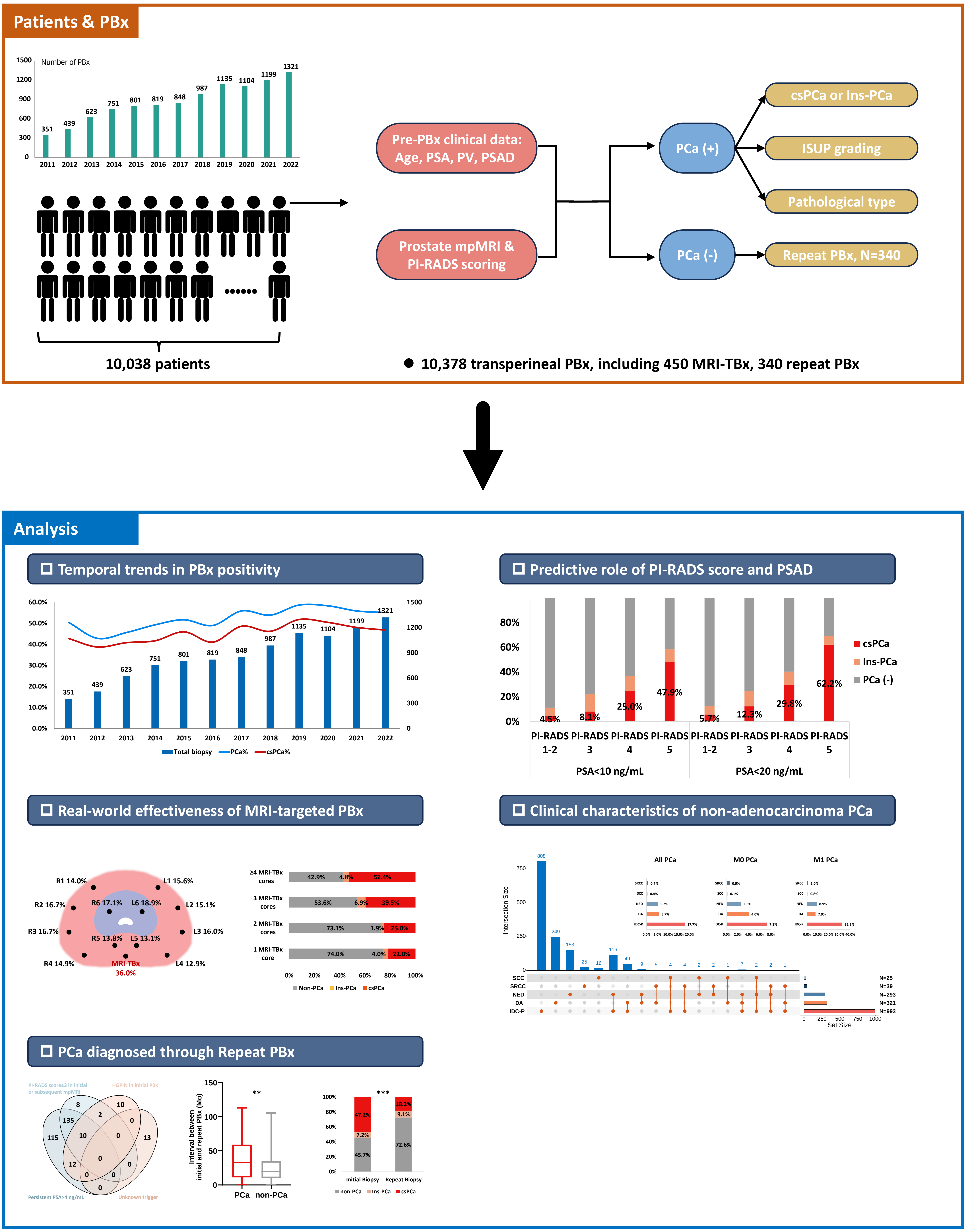

Figure S1. The overall study design

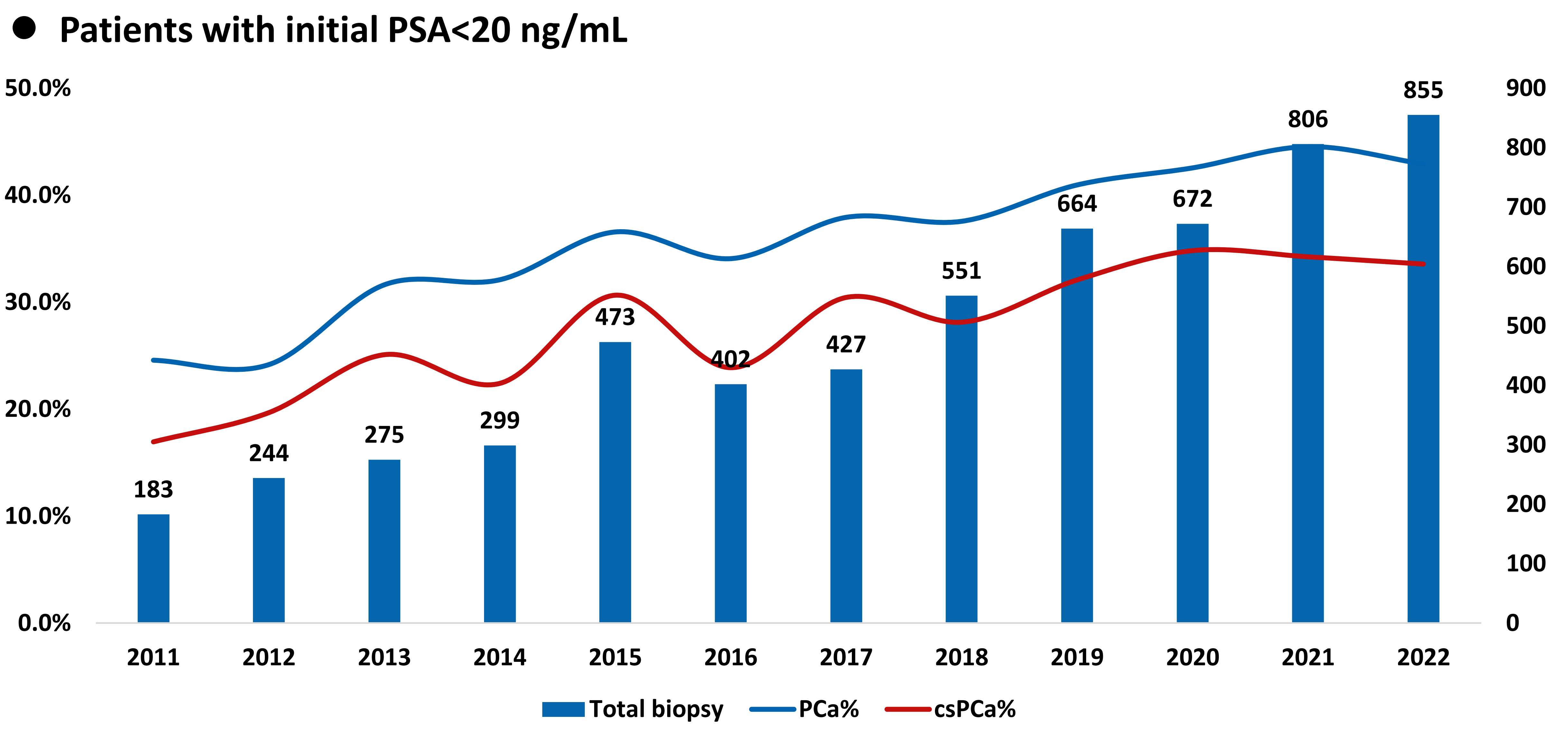

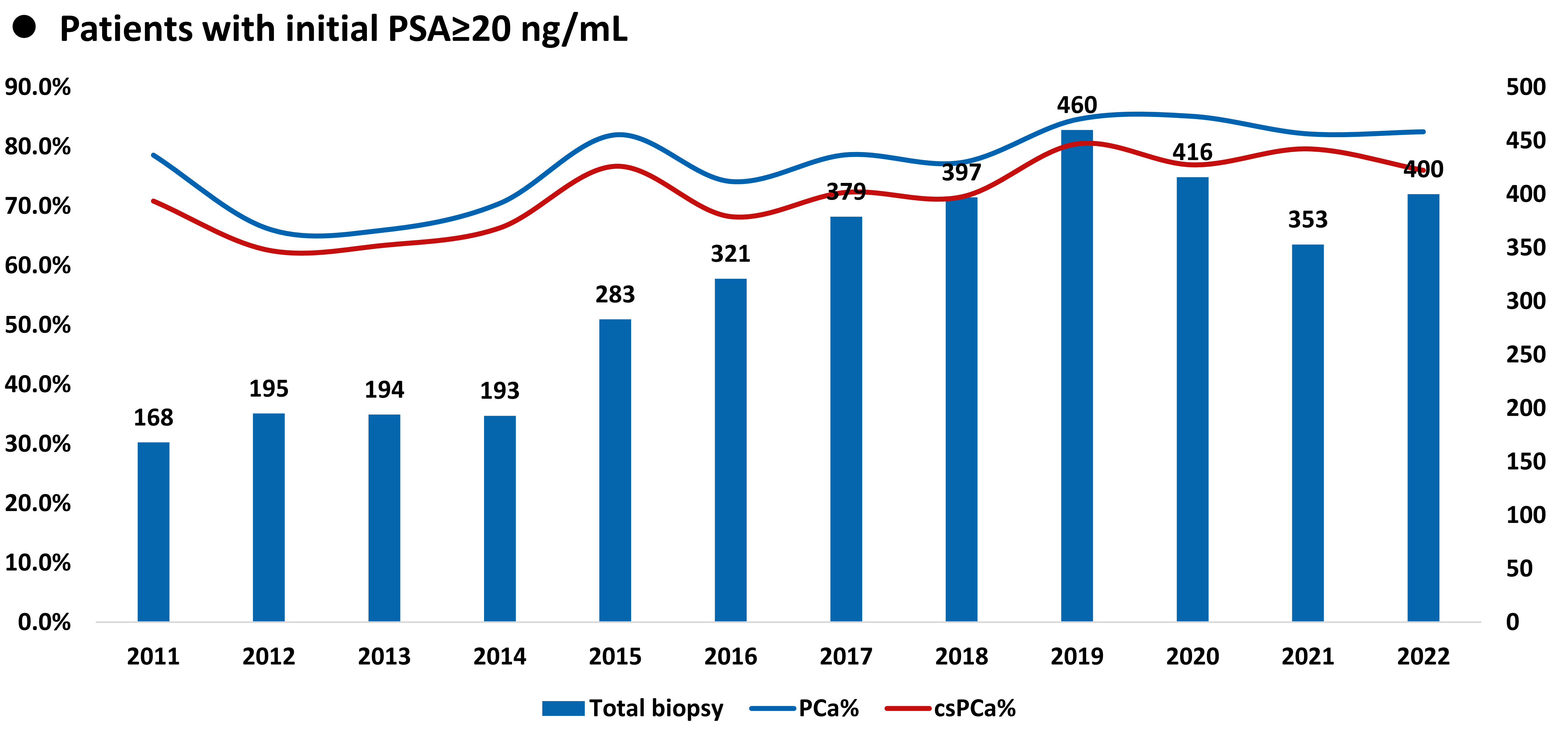

Figure S2. Temporal trends in clinical characteristics of prostate cancer classified by pre-biopsy PSA levels. PSA: prostate specific antigen; PCa: prostate cancer; csPCa: clinically significant prostate cancer

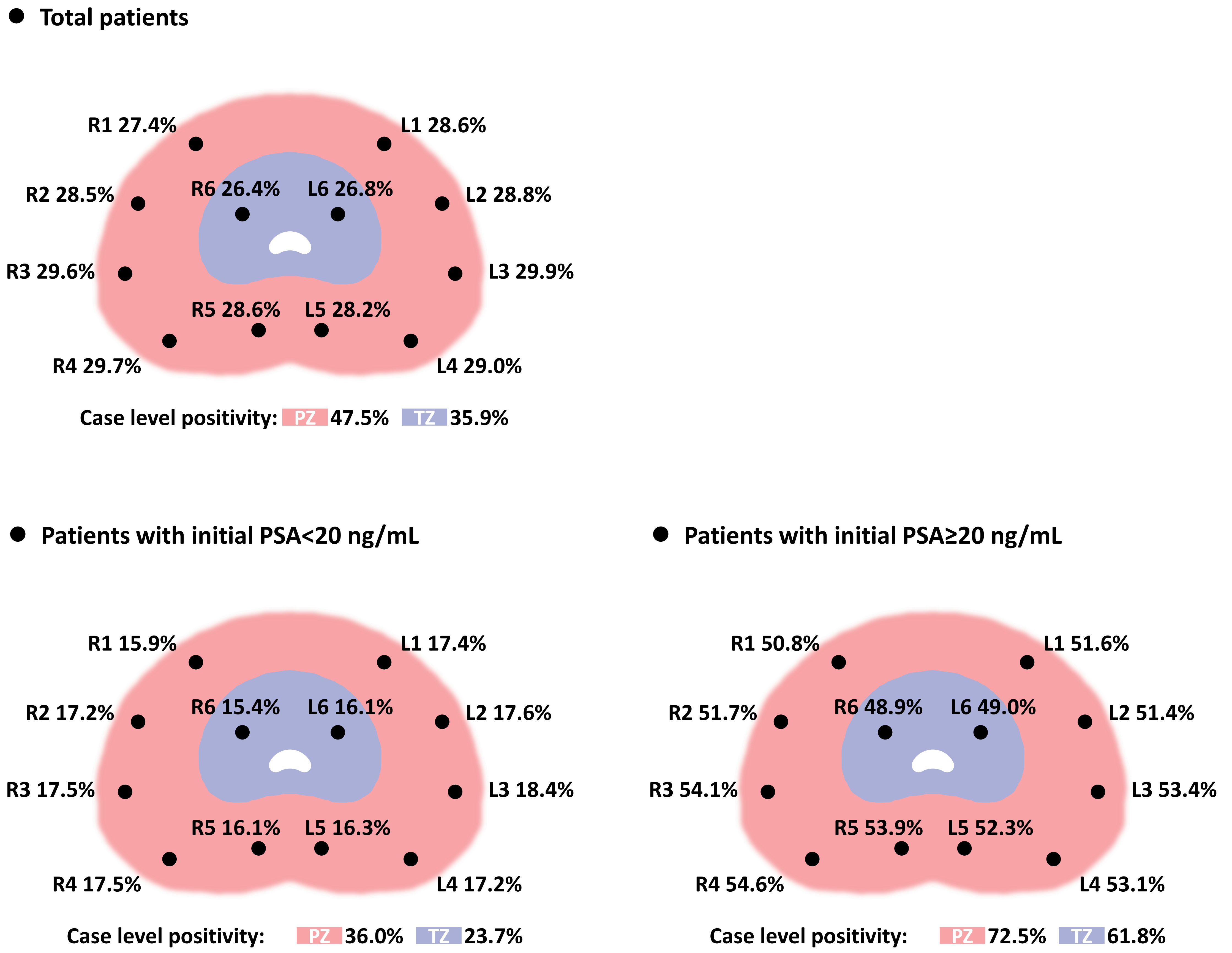

Figure S3. Core-level and case-level positivity rates for men undergoing standard 12-core systematic biopsies. PSA: prostate specific antigen; PZ: peripheral zone; TZ: transition zone

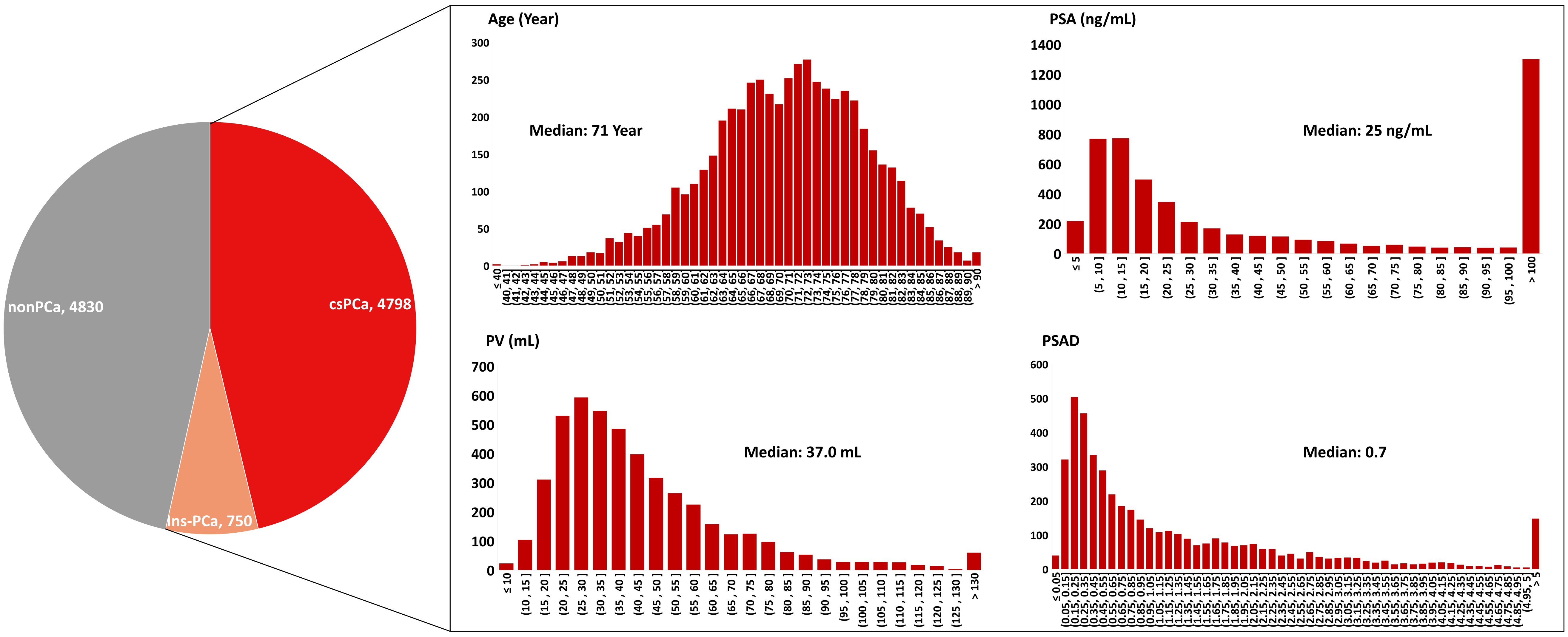

Figure S4. The distribution of age, PSA, PV, and PSAD among patients with PCa. PSA: prostate specific antigen; PV: prostate volume; PSAD: prostate specific antigen density; PCa: prostate cancer

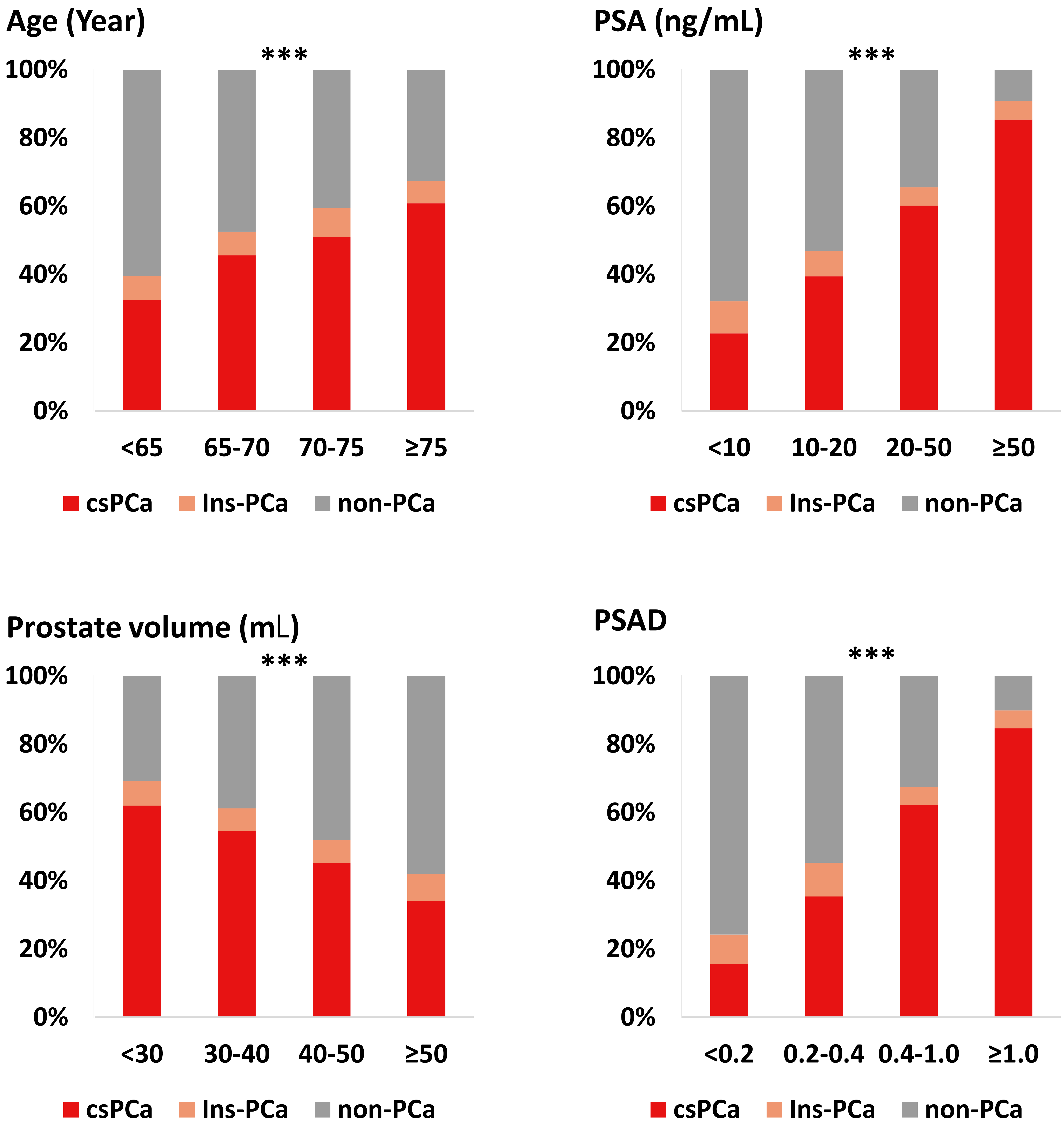

Figure S5. The value of age, baseline PSA levels, prostate volume, and PSAD in predicting the presence of csPCa and Ins-PCa.
csPCa: clinically significant prostate cancer; Ins-PCa: insignificant prostate cancer; PSA: prostate specific antigen; PSAD: prostate specific antigen density. *** P<0.001

A.

M0 PCa	2011	2012	2013	2014	2015	2016	2017	2018	2019	2020	2021	2022
Median Age (Year)	72	72	71	71	70	70	69	71	69	70	69	68
Median PSA (ng/mL)	14.91	15.63	11.41	11.78	14.005	14.38	16.52	15.23	13.77	12.1	10.4	10.2

B.

M1 PCa	2011	2012	2013	2014	2015	2016	2017	2018	2019	2020	2021	2022
Median Age (Year)	72	72	72.5	73	71	73	72	74	71	73	71	73
Median PSA (ng/mL)	≥100	≥100	≥100	≥100	≥100	≥100	≥100	≥100	≥100	≥100	≥100	≥100

C.

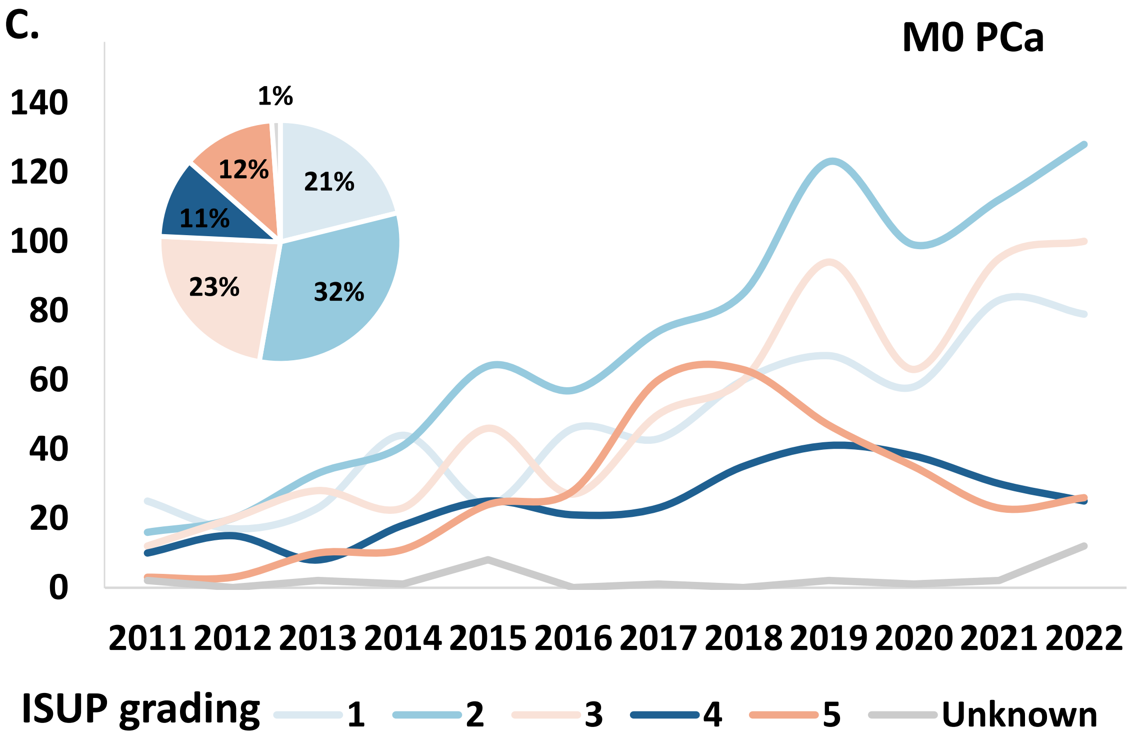

D.

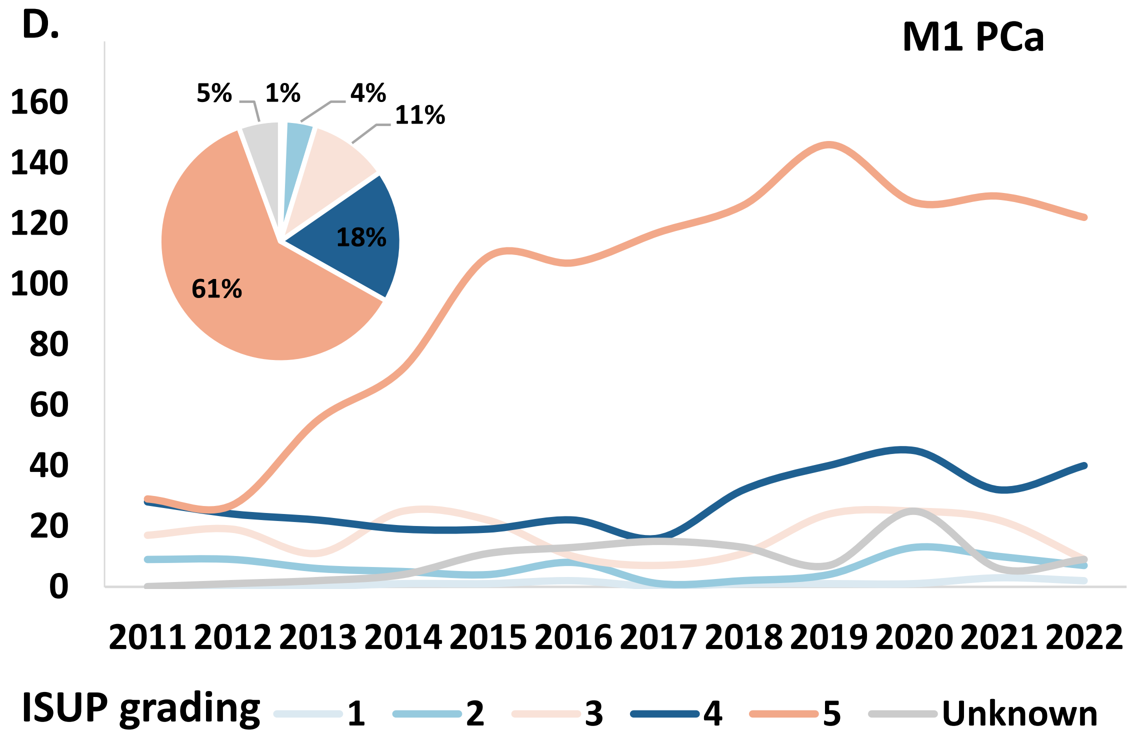

Figure S6. Temporal trends in clinical characteristics of prostate cancer, categorized by M stage. A, B: The temporal changes in the median age and PSA levels for patients diagnosed with M0 (C) and M1 (D) PCa. C, D: The temporal changes in the ISUP grading for patients diagnosed with M0 (C) and M1 (D) PCa.
PSA: prostate specific antigen; ISUP: International Society of Urological Pathology; PCa: prostate cancer

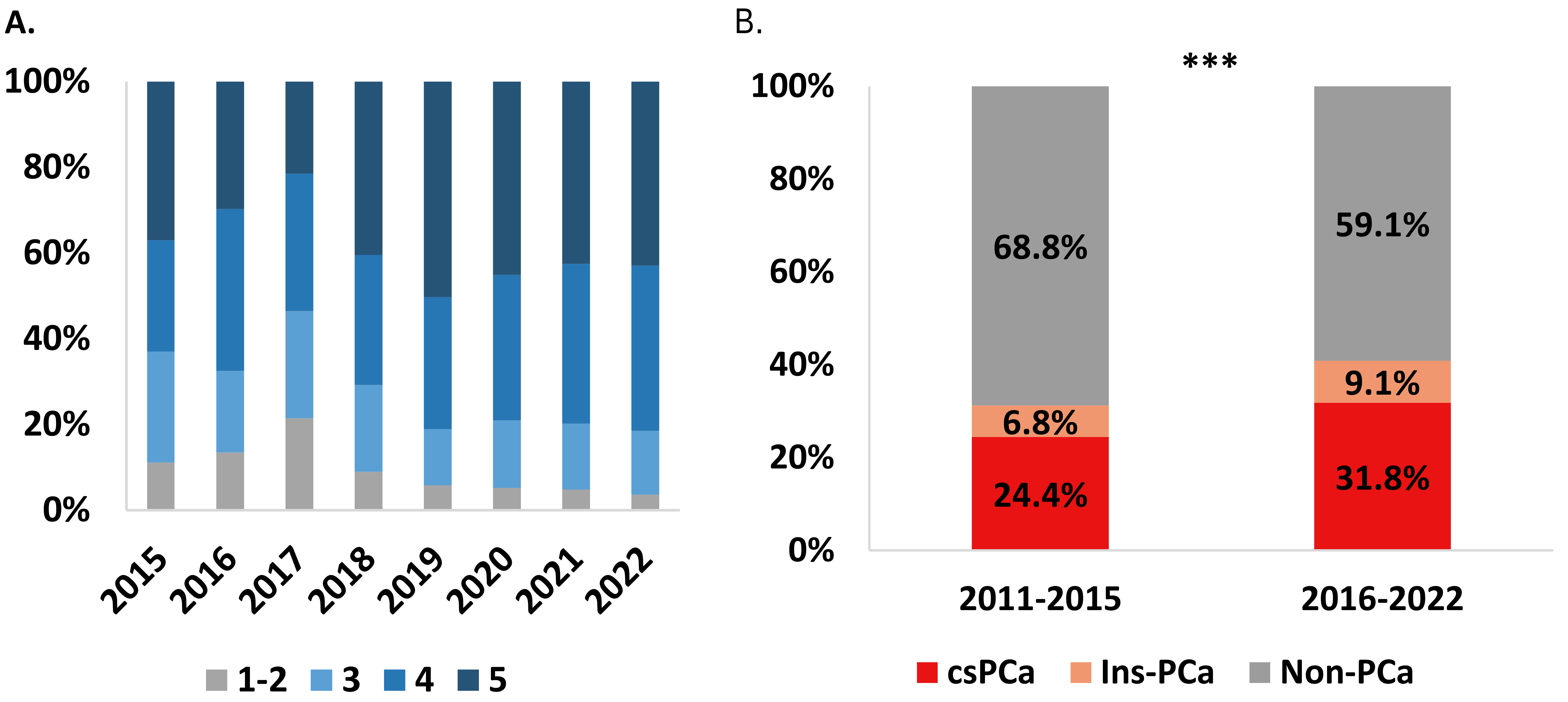

Figure S7. The comparison of PCa detection rate before and after the use of PI-RADS scoring. A. The distribution changes of PI-RADS scores in our center over years. B. The comparison of PCa detection rate before (2011-2015) and after (2016-2022) the use of PI-RADS scoring. PCa: prostate cancer; csPCa: clinically significant prostate cancer; Ins-PCa: insignificant prostate cancer. *** P<0.001

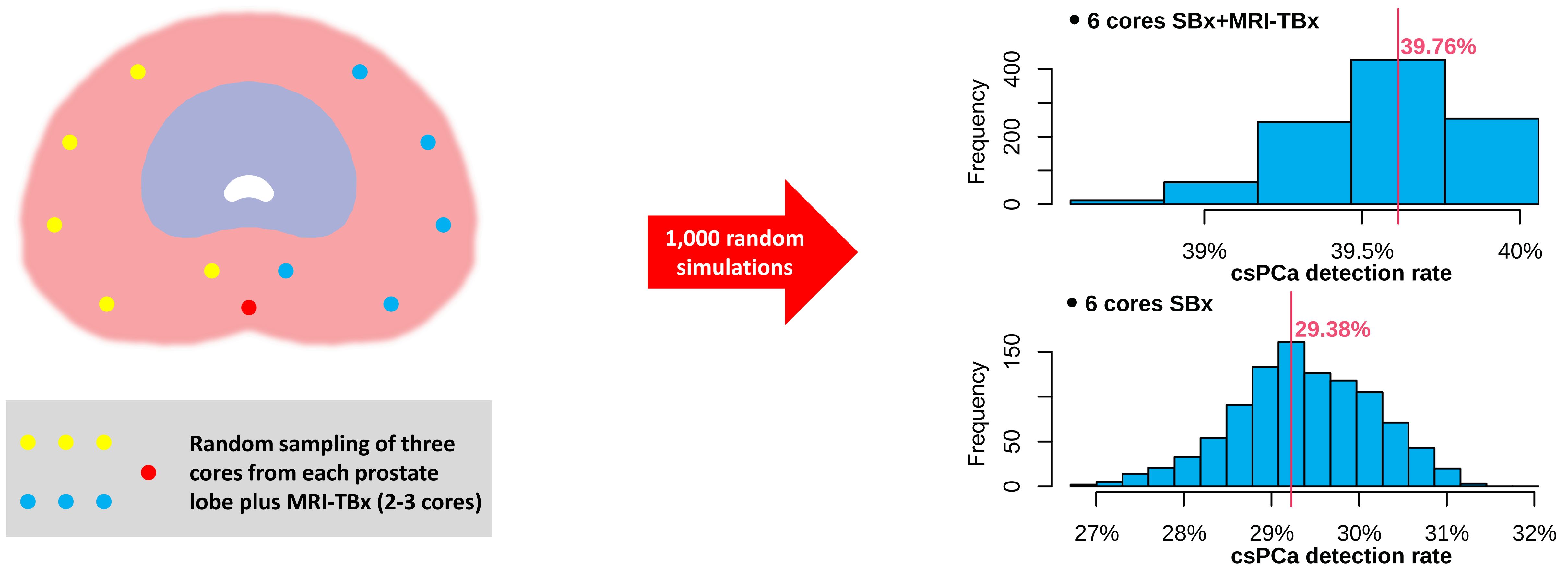

Figure S8. Schematic diagram depicting the detection rate of csPCa using a random 6 -core PBx, with or without the addition of MRI-TBx. csPCa: clinically significant prostate cancer; SBx: systematic biopsy; MRI-TBx: MRI-targeted prostate biopsy

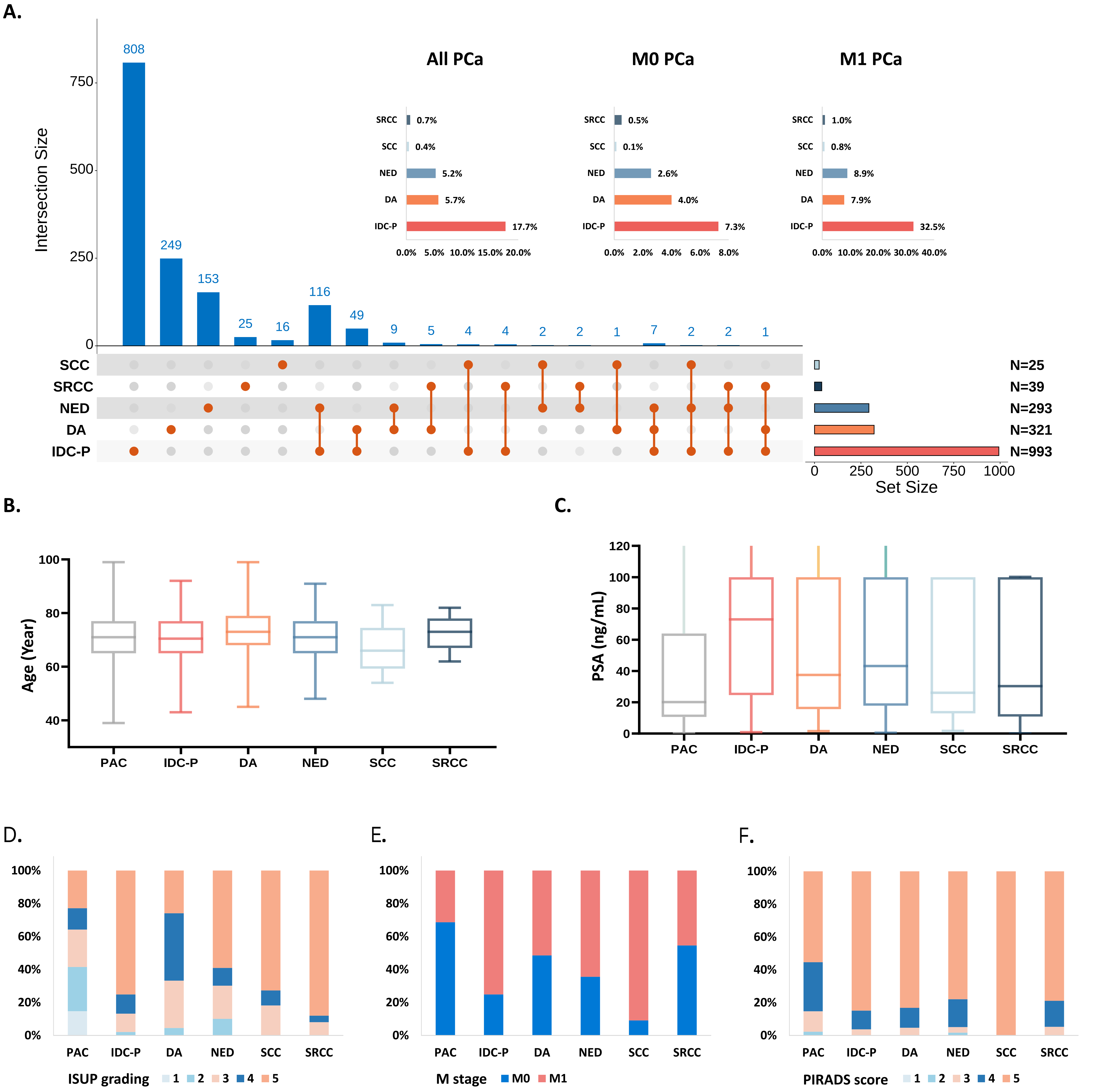

Figure S9. The detection rate and clinical characteristics of non-adenocarcinoma prostate cancer

A. The detection rate of various non-AC PCa. B-F. Comparison of age at diagnosis (B), baseline PSA levels (C), ISUP grading (D), M stage (E), and PI-RADS scoring (F) of tumors among patients with various pathological types of PCa.

Non-AC: non-adenocarcinoma; PCa: prostate cancer; PSA: prostate specific antigen; ISUP: International Society of Urological Pathology; PI-RADS: Prostate Imaging-Reporting and Data System

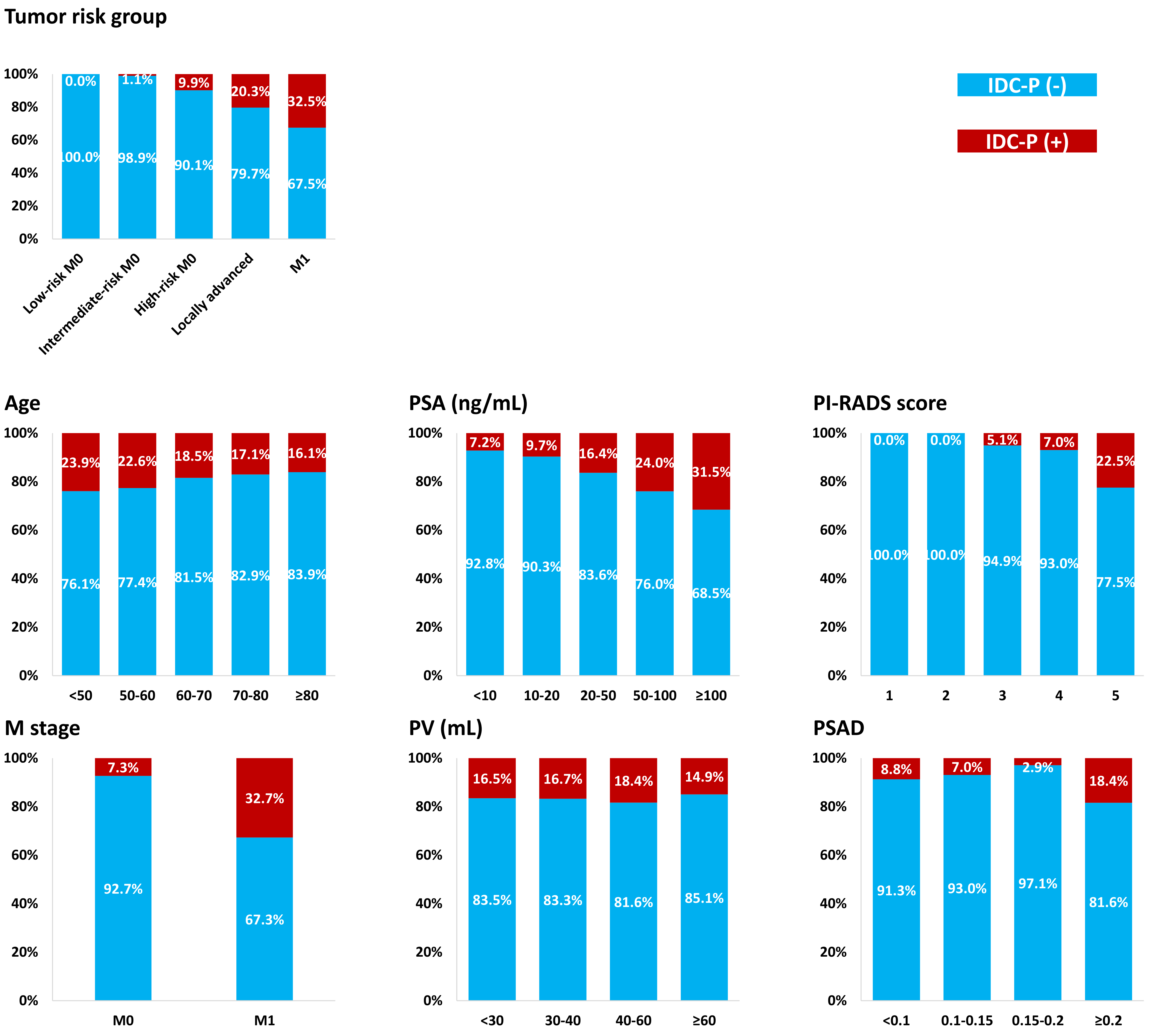

Figure S10. Detection rates of IDC-P in patients with varying risk groups and different baseline characteristics. IDC-P: Intraductal carcinoma of the prostate

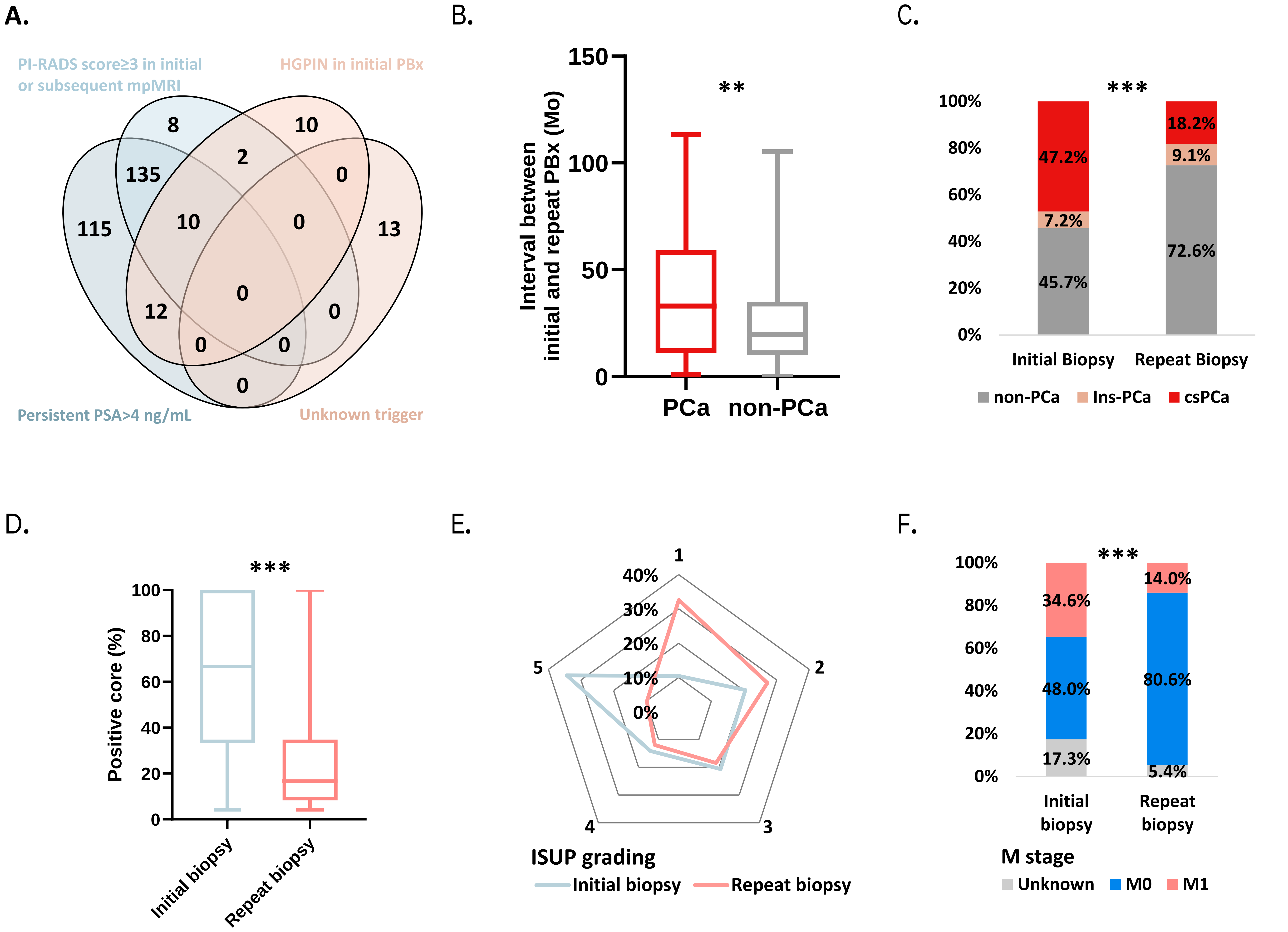

Figure S11. The clinicopathological features of prostate cancer diagnosed in repeat biopsy.

A. Reasons for repeat PBx. B. The comparison of time interval between initial and repeat PBx in men with and without PCa diagnosis in repeat PBx. C. The detection rate of csPCa and Ins-PCa for individuals undergoing initial and repeat PBx. D. The comparison of positive cores of PCa between initial and repeat PBx. E. The comparison of ISUP grading of PCa between initial and repeat PBx. F. The comparison of M stage of PCa between initial and repeat PBx.

PBx: prostate biopsy; PCa: prostate cancer; csPCa: clinically significant prostate cancer; Ins-PCa: insignificant prostate cancer; PBx: prostate biopsy; ISUP: International Society of Urological Pathology

*<0.05; **<0.01; ***<0.001
