# Supplementary material for: Decade-long insights into transperineal prostate biopsy in a West China population: temporal trend, targeted and repeat biopsies, and pathological characterization: a comparative study – retrospective cohort
Source: Int J Surg. 2024 Oct 25;111(1):1636–41. doi: 10.1097/JS9.0000000000002122 (PMC11745631; doi:10.1097/JS9.0000000000002122)
Supplement: Supplementary file 2 [file js9-111-1636-s002.docx]

**Table 1. Pre-biopsy characteristics of the total patients and those diagnosed with csPCa, Ins-PCa, or any PCa.**

|  | **Total biopsy**  **N=10378** | **csPCa**  **N=4798** | **Ins-PCa**  **N=750** | **All PCa**  **N=5548** |
| --- | --- | --- | --- | --- |
| **Age (Year)** |  |  |  |  |
| **<50** | 242 (2.33%) | 39 (0.81%) | 7 (0.93%) | 46 (0.83%) |
| **50-60** | 1247 (12.02%) | 375 (7.82%) | 93 (12.40%) | 468 (8.44%) |
| **60-70** | 3826 (36.87%) | 1555 (32.41%) | 271 (36.13%) | 1826 (32.91%) |
| **70-80** | 3863 (37.22%) | 2067 (43.08%) | 302 (40.27%) | 2369 (42.70%) |
| **≥80** | 1200 (11.56%) | 762 (15.88%) | 77 (10.27%) | 839 (15.12%) |
| **PSA (ng/mL)** |  |  |  |  |
| **<10** | 3085 (29.73%) | 688 (14.34%) | 291 (38.80%) | 979 (17.65%) |
| **10-20** | 2766 (26.65%) | 1063 (22.16%) | 208 (27.73%) | 1271 (22.91%) |
| **20-50** | 1689 (16.27%) | 1002 (20.88%) | 91 (12.13%) | 1093 (19.70%) |
| **50-100** | 700 (6.75%) | 540 (11.25%) | 27 (3.60%) | 567 (10.22%) |
| **≥100** | 1370 (13.20%) | 1219 (25.41%) | 84 (11.20%) | 1303 (23.49%) |
| **Unknown** | 768 (7.40%) | 286 (5.96%) | 49 (6.53%) | 335 (6.04%) |
| **PV (ml)** |  |  |  |  |
| **<30** | 2265 (21.83%) | 1396 (29.10%) | 165 (22.00%) | 1561 (28.14%) |
| **30-40** | 1697 (16.35%) | 911 (18.99%) | 122 (16.27%) | 1033 (18.62%) |
| **40-60** | 2441 (23.52%) | 1026 (21.38%) | 178 (23.73%) | 1204 (21.70%) |
| **≥60** | 2267 (21.84%) | 688 (14.34%) | 175 (23.33%) | 863 (15.56%) |
| **Unknown** | 1708 (16.46%) | 777 (16.19%) | 110 (14.67%) | 887 (15.99%) |
| **PSAD** |  |  |  |  |
| **<0.1** | 790 (7.61%) | 92 (1.92%) | 68 (9.07%) | 160 (2.88%) |
| **0.1-0.15** | 877 (8.45%) | 127 (2.65%) | 74 (9.87%) | 201 (3.62%) |
| **0.15-0.2** | 840 (8.09%) | 161 (3.36%) | 78 (10.40%) | 239 (4.31%) |
| **≥0.2** | 5917 (57.01%) | 3575 (74.51%) | 404 (53.87%) | 3979 (71.72%) |
| **Unknown** | 1954 (18.83%) | 843 (17.57%) | 126 (16.80%) | 969 (17.47%) |
| **PI-RADS score** |  |  |  |  |
| **1** | 21 (0.20%) | 7 (0.15%) | 3 (0.40%) | 10 (0.18%) |
| **2** | 197 (1.90%) | 14 (0.29%) | 13 (1.73%) | 27 (0.49%) |
| **3** | 572 (5.51%) | 96 (2.00%) | 62 (8.27%) | 158 (2.85%) |
| **4** | 1286 (12.39%) | 449 (9.36%) | 124 (16.53%) | 573 (10.33%) |
| **5** | 1627 (15.68%) | 1227 (25.57%) | 94 (12.53%) | 1321 (23.81%) |
| **Unknown** | 6675 (64.32%) | 3005 (62.63%) | 454 (60.53%) | 3459 (62.35%) |

Ins-PCa: Insignificant prostate cancer; csPCa: clinically significant prostate cancer; PSA: prostate specific antigen; PV: prostate volume; PSAD: prostate specific antigen density; PI-RADS: prostate imaging-reporting and data system
